# Are Gastrointestinal Symptoms Important in Patients with COVID-19?

**DOI:** 10.5152/tjg.2023.22160

**Published:** 2023-03-01

**Authors:** Emine Büküm, Ayşe Oğuz Ayarcı, Kadir Güler

**Affiliations:** 1 Departmant of General Surgery, Bursa City Hospital, Bursa, Turkey; 2 Department of Infectious Diseases, Bursa City Hospital, Bursa, Turkey

**Keywords:** Abdominal pain, COVID-19, diarrhea, nausea, prognosis

## Abstract

**Background:**

Our study aimed to present the general characteristics of patients in our country with coronavirus disease 2019 (COVID-19) having gastrointestinal system symptoms and the effects of gastrointestinal system symptoms on prognosis in the light of literature.

**Methods:**

This retrospective single-center study included patients who tested positive for COVID-19 in polymerase chain reaction test and were treated as symptomatic inpatients between April 1, 2020, and May 15, 2020, at the Bursa City Hospital in Turkey.

**Results:**

In our study, 292 patients with positive COVID-19 polymerase chain reaction test and symptoms were included to investigate the effect of gastrointestinal system symptoms in COVID-19 patients. Patients with at least 1 gastrointestinal system symptom were named group 1, and patients with no gastrointestinal system symptoms were named group 2. Compared with group 1 (145 patients), group 2 (147 patients) had patients with significantly older age (*P* = .010) and significantly higher rates of intensive care unit admission (*P* = .023), intubation (*P* = .021), and exitus (*P* = .004). The white blood cell count (*P* = .001) and C-reactive protein (*P* = .001) values were significantly higher in group 2 than in group 1.

**Conclusion:**

COVID-19 is primarily a disease with respiratory symptoms, but gastrointestinal system symptoms are also seen. In our study, we found that patients with gastrointestinal system symptoms had a better prognosis. The reason for this may be the early hospitalization of the patients due to gastrointestinal system symptoms and the early initiation of treatment. However, comprehensive studies are still needed to elucidate this further.

**Main Points**

- Respiratory symptoms may be accompanied by gastrointestinal system (GIS) symptoms at different rates in coronavirus disease 2019. Sometimes patients may present with only GIS symptoms.
- Gastrointestinal involvement is seen in young patients and is associated with a good prognosis.
- Early hospitalization due to GIS symptoms and early initiation of treatment may have positively affected the prognosis of the disease.

## Introduction

The new coronavirus 2019 disease (COVID-19) initially appeared in Wuhan, China, in December 2019 and turned into a global pandemic affecting China first followed by the whole world. It was declared a pandemic by the World Health Organization on March 11, 2020. The disease spread rapidly all over the world, infected millions of people, and resulted in a large number of casualties.

Patients with COVID-19 mostly present with respiratory system symptoms, such as fever, cough, and shortness of breath. Although COVID-19 commonly causes respiratory symptoms, some patients also present with non-respiratory symptoms. Some of these symptoms are gastrointestinal system (GIS) symptoms, such as nausea, vomiting, abdominal pain, and diarrhea.^1^ In a study by Klopfenstein et al^2^ diarrhea was most frequently used as the fifth symptom.

The clinical picture of COVID-19 infection can range from mild to critical in terms of severity; a total of 81% of the patients had a mild illness, 14% had a severe illness, and 5% had a critical illness.^3^ Patients over the age of 65, as well as those with hypertension, diabetes mellitus, malignancy, chronic respiratory system diseases, and a history of cardiovascular disease, are at risk in terms of the severity of the disease.^4^ In a study including 204 cases based in China, it was seen that GIS symptoms were more pronounced as the severity of the disease increased.^5^ However, in another study in New York that included 150 patients, GIS symptoms were detected in 31 patients and no relationship was found between GIS symptoms and mortality, hospitalization rate, and intubation.^6^

Sometimes, COVID-19 may present only with GIS symptoms without showing any respiratory symptoms. Recent studies on COVID-19 indicate that there is a relationship between gastrointestinal damage and severe acute respiratory syndrome coronavirus-2 (SARS-CoV-2) infection.^7,8^

Angiotensin-converting enzyme 2 (ACE2), a zinc metallopeptidase, is a receptor commonly found on the surface of endothelial and epithelial cells. The SARS-CoV-2 virus enters the cell through the ACE2 receptors on the cell surface. After the virus binds to the ACE2 receptor and the ACE2-virus complex enters the target cell, the virus RNA is released into the cytoplasm and viral replication begins.^9^ Recent studies have shown that the ACE2 receptor required for cells infected with COVID-19 is highly expressed not only in the lungs but also in absorptive enterocytes in the ileum and colon.^10,11^ Angiotensin-converting enzyme 2 expression levels are much higher in the GIS than in the lungs.^12^ The GIS tropism of SARS-CoV-2 is also supported by evidence of high levels of calprotectin, a protein marker for intestinal inflammation.^13^ Although stomach acid can significantly reduce the lifespan of the virus, SARS-CoV-2 can potentially travel to the duodenum and distal small intestine. This makes the GIS a target for the virus.^14^

Severe acute respiratory syndrome coronavirus-2 was successfully isolated from stool samples of COVID-19 patients.^15,16^ In a study conducted with 74 patients, the respiratory samples remained positive for SARS-CoV-2 RNA for an average of 16.7 days from the onset of the symptoms in 41 (55%) of the patients whose stool samples were positive for SARS-CoV-2 RNA. The stool samples of the same patients, however, remained positive in polymerase chain reaction (PCR) tests for 27.9 days on average.^17^ It is of interest in terms of fecal–oral transmission that the stool samples of COVID-19 patients are positive even after the symptoms have completely disappeared. Moreover, it should be kept in mind that GIS involvement leads to higher viral load and/or prolonged viral transmission, allowing the virus to spread to other organs.^18^

In a study conducted with 59 patients with COVID-19 in Hong Kong, 9 patients (15.3%) were found to have stool PCR positivity. In the same study, patients without diarrhea had stool PCR positivity, although at a lower rate. The median viral load of RNA in the stool was higher in individuals with diarrhea compared with those without diarrhea (5.1 log_10_ copies/mL vs. 3.9 log_10_ copies/mL; *P* = .06).^19^ This indicates that the virus may be present in the GIS without causing diarrhea. In a patient with typical covid pneumonia on thorax CT and negative multiple nasopharyngeal and oropharyngeal PCR results, the presence of viral SARS-CoV-2 was confirmed by stool PCR alone.^20^ In a meta-analysis involving 8136 patients for the detection of SARS-CoV-2 using reverse transcriptase-PCR in different types of clinical specimens, the widely used nasopharyngeal PCR was found to have a moderate PR (positive reproductive value) of 45.5%. In the same meta-analysis, bronchoalveolar lavage had the highest PR (91.8%), followed by rectal swab (87.8%) and sputum (68.1%).^21^

Coronavirus disease 2019 may not be initially considered as the diagnosis in patients who are admitted to the hospital with only GIS symptoms. For this reason, there is usually a delay in the diagnosis of the disease and time until the first respiratory symptoms appear. This makes these patients a potential source of viral transmission. Therefore, GIS symptoms are particularly important in COVID-19.^22^

## Materials and Methods

The study was designed as a retrospective single-center study. The present study included patients who applied to the Bursa City Hospital in Turkey on April 1, 2020, and May 15, 2020, whose nasopharyngeal samples tested positive for SARS-CoV-2 in the PCR test, who had at least 1 COVID-19 symptom, and who were hospitalized or admitted to the intensive care unit (ICU) for treatment. Pregnant patients and patients aged <18 years were excluded from the study. A total of 292 patients who met the inclusion criteria were included in the study. The patients’ files were reviewed to obtain and record data regarding their demography, complaints, hospitalization information, chest computed tomography (CT), and laboratory results. Official approval for the study was received from the Ministry of Health on May 19, 2022, with the number 2020-05-15T15_27_14 and from the Bursa City Hospital Ethics Committee with the number 2020-4/4 on July 29, 2020. The study was conducted in accordance with the Declaration of Helsinki.

Patients with at least 1 GIS symptom were included in group 1 and those without any GIS symptoms in group 2. Diarrhea, abdominal pain, nausea, and vomiting were recorded as GIS symptoms. Defecation at least 3 times a day was defined as diarrhea. Fever, weakness, muscle pain, headache, cough, respiratory distress, sore throat, loss of appetite, and an inability to taste and smell were recorded as non-gastrointestinal symptoms.

### Statistical Analysis

All analyses were performed on Statistical Package for the Social Sciences software version 21 (IBM Corp.; Armonk, NY, USA). Kolmogorov–Smirnov test was used for normality control. Data were presented as mean ± standard deviation or median value (first quarter to third quarter) for normally distributed continuous variables and as frequency (percentage) for categorical variables. Normally distributed variables were analyzed using the independent samples *t*-test. Variables with non-normal distribution were analyzed using the Mann–Whitney *U* test. Categorical variables were analyzed using Chi-square tests or Fisher’s exact tests. Values of *P* < .05 were considered statistically significant.

## Results

We included 292 patients who tested positive for COVID-19 in the PCR test and presented with at least 1 COVID-19 symptom. Of these, 144 (49.32%) patients were male and 148 (50.68%) were female. The mean age was 51.28 ± 16.81 (19-95). The most common comorbidities were hypertension (27.40%), diabetes mellitus (16.44%), heart disease (12.33%), and 169 (56.1%) patients did not have any chronic disease (Table 1).

**Figure 1.**
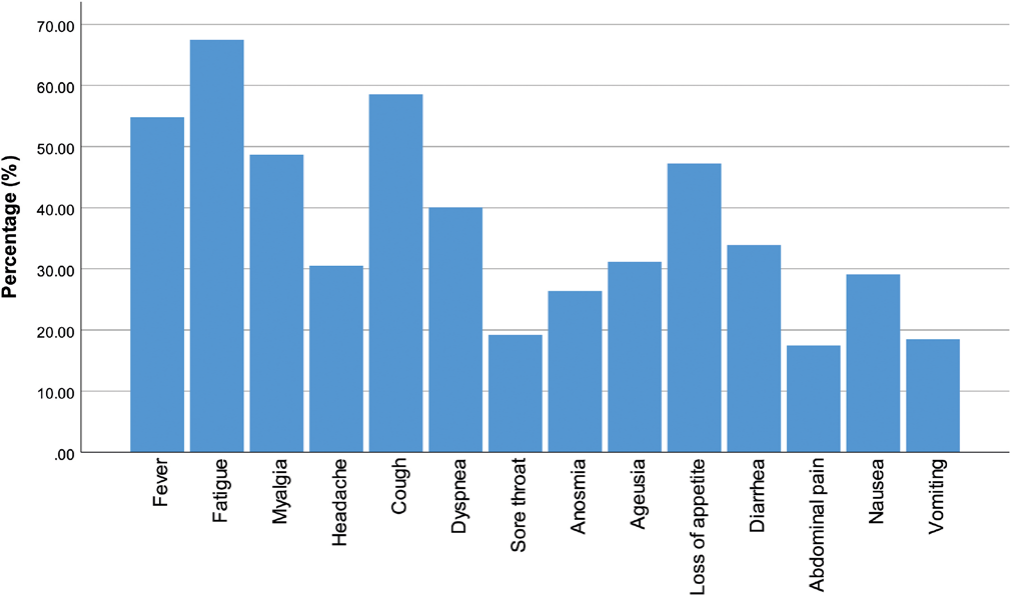
General symptoms.

The most common symptoms were fatigue (197, 67.47%), cough (171, 58.56%), and fever (160, 54.79%). In addition, 142 (48.63%) patients had myalgia, 138 (47.26%) had loss of appetite, 117 (40.07%) had shortness of breath, 99 (33.90%) had diarrhea, 91 (31.16%) had dysgeusia, 89 (30.48%) had headache, 85 (29.11%) had nausea, 77 (26.37%) had anosmia, 56 (19.18%) had sore throat, 54 (18.49%) had vomiting, and 51 (17.47%) had abdominal pain (Figure 1).

Computed tomography of the chest was available for all patients included in the study. A total of 232 (79.45%) patients had chest CT findings associated with COVID-19, 59 (20.21%) were admitted to the ICU, 24 (8.22%) were intubated, and 25 (8.56%) died. The mean hospitalization duration of all patients was 11 (2-74) days (Table 1).

**Figure 2.**
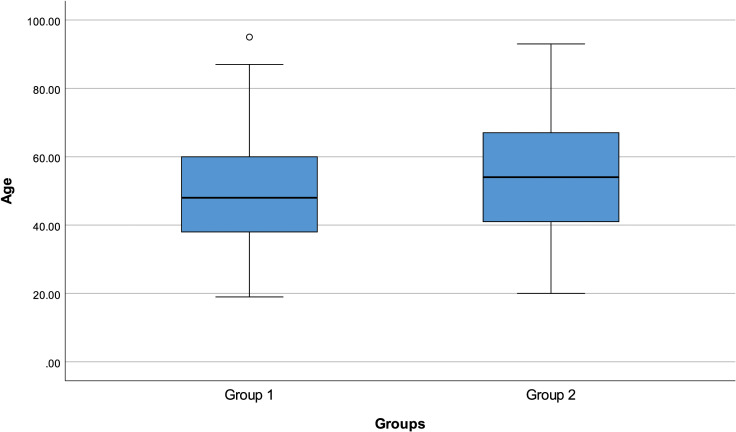
Group 1 and group 2 age distribution.

**Figure 3.**
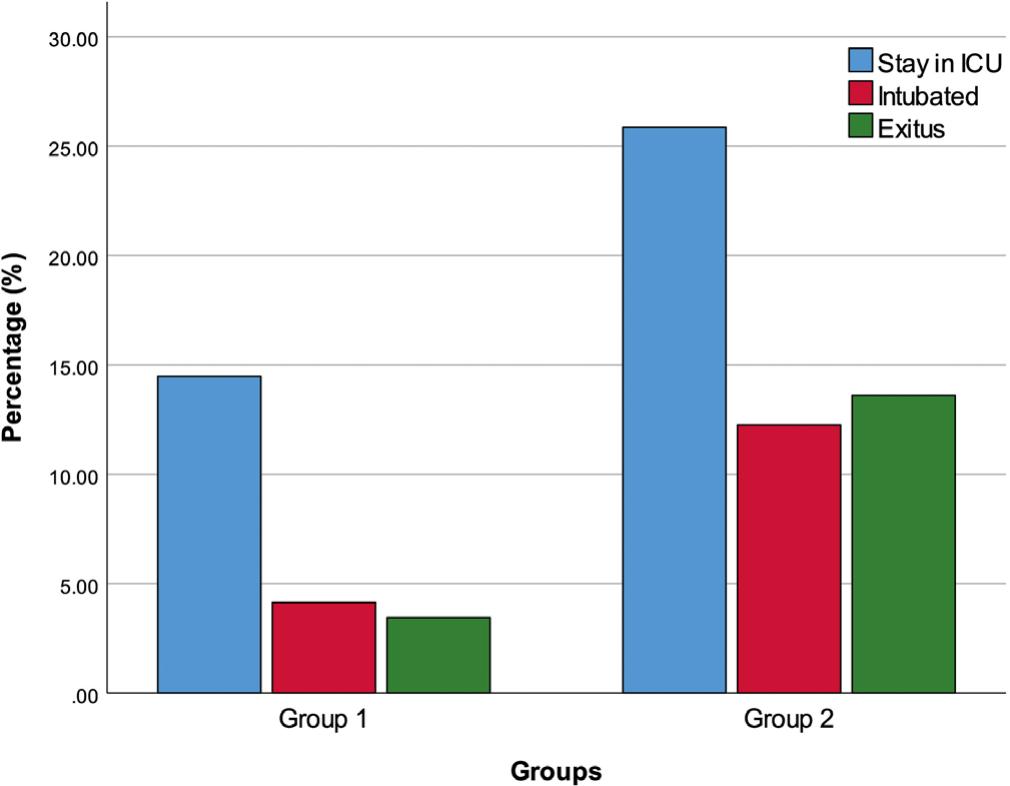
Group 1 and group 2 stay in ICU, intubation, and exitus. ICU, intensive care unit.

We divided the patients into 2 groups as group 1 (145 patients) with at least 1 GIS symptom and group 2 (147 patients) without any GIS symptoms. The patient’s age was significantly higher in group 2 compared with group 1 (*P* = .010) (Figure 2). Additionally, the ICU admission rate (*P* = .023), the percentages of intubation (*P* = .021), and exitus (*P* = .004) were significantly higher in group 2 than in group 1 (Figure 3).

White blood cell (WBC) (*P* = .001) and C-reactive protein (CRP) (*P* = .001) values were significantly higher in group 2 patients compared with group 1 patients. There was no significant difference between the 2 groups in terms of sex, comorbidities, chest CT findings, length of hospital stay, and number of days spent in the ICU, as well as lymphocyte, hemoglobin, and values of aspartate and alanine aminotransferases (Table 2).

The demographic data and clinical characteristics of the patients were compared in terms of each GIS symptom. A total of 99 (33.90%) patients had diarrhea. The mean age of the patients with diarrhea was 45.30 ± 13.49. The age of patients with diarrhea was significantly lower than those without diarrhea (*P* < .001). There was no significant difference between patients with and without diarrhea in terms of sex, CT findings, ICU admission, intubation rate, exitus rate, length of stay in the ICU, and hospitalization duration (Table 3).

Fifty-one (17.47%) patients had abdominal pain. The mean age of patients with abdominal pain was 46.65 ± 15.46. Age was significantly lower in patients with abdominal pain than those without abdominal pain (*P* = .030). The percentage of men among those with abdominal pain was significantly lower than among those without abdominal pain (*P* = .040). There was no significant difference between patients with and without abdominal pain in terms of CT findings, ICU admission, intubation rate, exitus rate, length of stay in the ICU, and duration of hospitalization (Table 3).

Eighty-five (29.11%) patients had nausea. The mean age of the patients with nausea was 47.14 ± 16.49. The age of patients with nausea was significantly lower than those without nausea (*P* = .007). The percentage of men among those with nausea was significantly lower than those without nausea (*P* = .011). There was no significant difference between patients with and without nausea in terms of CT findings, ICU admission, intubation rate, exitus rate, length of stay in the ICU, and duration of hospitalization (Table 3).

Fifty-four (18.49%) patients had vomiting. The mean age of patients with vomiting was 51.35 ± 18.45. There was no significant difference between patients with and without vomiting in terms of age, sex, CT findings, ICU admission, intubation rate, exitus rate, length of stay in the ICU, and duration of hospitalization (Table 3).

## Discussion

Although COVID-19 presents with respiratory symptoms, a considerable number of patients may also present with GIS symptoms. Gastrointestinal system involvement is a known feature in coronavirus infections, and GIS symptoms have been observed to predominate in SARS and Middle-east respiratory syndrome outbreaks in the past.^23^ A meta-analysis that investigated the general characteristics of COVID-19 in China and included the data of 1099 patients found that the most common symptoms were cough (67.8%), fever (43.8%), and fatigue (38.1%).^24^ In our study, the most common symptoms were fatigue (67.47%), cough (58.56%), and fever (54.79%). Fatigue was pronounced in our population. Additionally, the mean age of the patients in the same meta-analysis was 47.0, whereas it was 51.28 in our study.

Our study aimed to investigate the general characteristics of GIS involvement and its relationship with the prognosis of the disease. Studies investigating GIS symptoms report varying rates of symptoms. A meta-analysis showed that GIS symptoms were present in 1 out of 5 patients.^25^ According to another meta-analysis, although gastrointestinal symptoms were reported in 15% of COVID-19 patients and liver damage in 19% of patients, approximately 10% of patients in the same study presented with gastrointestinal symptoms alone without any respiratory complaints. It was observed that as the severity of the disease increased, digestive symptoms and liver damage became more pronounced.^26^ In our study, the number of patients with at least 1 GIS symptom was 145 (0.496%) and the number of patients without GIS symptoms was 147 (0.503%), and the number of patients with GIS symptoms was higher than that observed in literature. This higher number may be attributed to a tendency of hospitalizing as many PCR-positive patients as possible in the country for ensuring isolation and preventing the spread during the period of the study.

Previous studies report varying results on whether there is a relationship between GIS symptoms and disease severity. A study investigating the GIS findings of hospitalized COVID-19 patients found no significant difference between patients with at least 1 GIS symptom and no GIS symptom in terms of hospitalization duration as well as intubation and exitus rates.^6^ Another meta-analysis concluded that GIS symptoms and the presence of elevated liver enzymes were not related to mortality or ICU admission.^27^ A study by Avcı et al^28^ in Turkey found that the severity of COVID-19 and the presence of GIS symptoms were related to each other, concluding that the presence of GI symptoms results in a 7.2-fold increased probability of increased disease severity. In our study, there was no difference between the 2 groups in terms of hospitalization and ICU stay durations. However, the rates of admission to the ICU (*P* = .023), intubation (*P* = .021), and exitus (*P* = .004) in group 2 patients were significantly higher than those in group 1 patients. In addition, it was observed that the mean ages of the group 1 patients was lower than those in group 2 patients (*P* = .010). When we looked at each of the GIS symptoms separately, the mean ages of the patients with diarrhea, abdominal pain, and nausea were significantly lower. When laboratory findings were compared, it was observed that the WBC and CRP values of group 1 patients were significantly lower than that of group 2 patients. There was no difference between the 2 groups in terms of comorbidities. This study concluded that patients with GIS symptoms had less disease severity and better prognosis. However, more comprehensive studies are needed on this subject.

A study investigating the clinical characteristics of COVID-19 patients with GIS symptoms reported that as the severity of the disease increased, the digestive symptoms became more pronounced, which was attributed to a lack of initial presentation of typical respiratory symptoms in patients with extrapulmonary symptoms leading to a delay in the diagnosis and worsening of prognosis.^5^ In our study, it was observed that patients with GIS symptoms had a better prognosis. At the time of the study, all COVID-19 PCR-positive patients in our country were hospitalized for isolation, regardless of their clinic. In our hospital, patients with mild respiratory symptoms were hospitalized only because they were PCR-positive. No studies have been conducted between the viral load of the patients and their clinic. Antiviral treatment, oxygen support and, if necessary, additional treatments were started in line with current information. Early initiation of treatment may have prevented clinical worsening in patients with GIS symptoms. This may explain the better prognosis of patients hospitalized with GIS symptoms in our study.

In a meta-analysis by Weibiao et al^29^ patients with abdominal pain were found to be associated with a 2.8-fold increased severity of illness. The same meta-analysis also found that the relationship between diarrhea and the severity of the disease was regionally different, and the relationship between nausea or vomiting and the severity of the disease was limited. In our study, when GIS symptoms were examined 1 by 1, no significant difference was observed in terms of the prognosis of the disease.

Pregnant women and patients under the age of 18 were not included in the study because the approach was different. In addition, since the study belongs to the early period of the pandemic, the tendency of patients to be hospitalized early for isolation at that time may have caused the number of patients with GIS symptoms to be high. These are the limitations of our study, but we think that they do not affect the results too much.

In conclusion, there are few studies in Turkey evaluating the frequency of GIS symptoms and their relation with prognosis in COVID-19 patients. Therefore, there are limited data in this regard in Turkey. In COVID-19, respiratory symptoms can be accompanied by GIS symptoms at different rates. Sometimes patients may present only with GIS symptoms. Therefore, COVID-19 infection should be considered as a diagnosis in patients with GIS symptoms during the pandemic, and clinicians should be aware that COVID-19 patients may also present only with diarrhea and nausea. This may aid in early diagnosis and early treatment of the disease and prevention of transmission. In addition, it is important to take measures to prevent transmission by raising awareness regarding this among healthcare professionals. We believe that our study will contribute to the existing data on Turkey and in literature.

## Figures and Tables

**Table 1.** General Findings of the Patients

	Age	51.28 ± 16.81
Gender	Male	144 (49.32%)
	Female	148 (50.68%)
Comorbidities	Hypertension	80 (27.40%)
	Diabetes mellitus	48 (16.44%)
	Heart diseases	36 (12.33%)
	Renal diseases	11 (3.77%)
	Malignancy	5 (1.71%)
	Neurologic diseases	14 (4.79%)
	COPD	22 (7.53%)
	Rheumatic diseases	9 (3.08%)
Symptoms	Fever	160 (54.79%)
	Fatigue	197 (67.47%)
	Myalgia	142 (48.63%)
	Headache	89 (30.48%)
	Cough	171 (58.56%)
	Dyspnea	117 (40.07%)
	Sore throat	56 (19.18%)
	Anosmia	77 (26.37%)
	Ageusia	91 (31.16%)
	Loss of appetite	138 (47.26%)
	Diarrhea	99 (33.90%)
	Abdominal pain	51 (17.47%)
	Nausea	85 (29.11%)
	Vomiting	54 (18.49%)
Groups	Group 1	145 (49.66%)
	Group 2	147 (50.34%)
CT, ICU, Intubation	CT findings	232 (79.45%)
	Stay in ICU	59 (20.21%)
	Intubated	24 (8.22%)
Outcome	Discharged	267 (91.44%)
	Exitus	25 (8.56%)
Length of stay	Length of stay in ICU (days)	11 (4-15)
	Length of stay in hospital (days)	11 (6-16)
Laboratory finding	WBC (×1000)	5.92 (4.54-7.55)
	Lymphocyte (×1000)	1.33 (0.95-1.84)
	Hemoglobin	13.39 ± 1.84
	AST	23.25 (18.00-33.10)
	ALT	19 (13-29)
	CRP	14.10 (4.50-43.25)

COPD, chronic obstructive pulmonary disease; CT, computed tomography; ICU, intensive care unit; WBC, white blood cell; AST, aspartate transaminase; ALT, alanine transaminase; CRP, C-reactive protein.

**Table 2.** Group 1 and Group 2 Comparison

		**Group 1 (n = 145)**	**Group 2 (n = 147)**	***P***
**Age**		48.74 ± 15.93	53.79 ± 17.32	**.010**
**Gender**	Male	65 (44.83%)	79 (53.74%)	.128
	Female	80 (55.17%)	68 (46.26%)	
**Comorbidities**	Hypertension	39 (26.90%)	41 (27.89%)	.953
	Diabetes mellitus	18 (12.41%)	30 (20.41%)	.092
	Heart diseases	17 (11.72%)	19 (12.93%)	.893
	Renal diseases	5 (3.45%)	6 (4.08%)	1.000
	Malignancy	1 (0.69%)	4 (2.72%)	.371
	Neurologic diseases	7 (4.83%)	7 (4.76%)	1.000
	COPD	13 (8.97%)	9 (6.12%)	.485
	Rheumatic diseases	5 (3.45%)	4 (2.72%)	.749
**CT, ICU, Intubation**	CT findings	112 (77.24%)	120 (81.63%)	.353
	Stay in ICU	21 (14.48%)	38 (25.85%)	**.023**
	Intubated	6 (4.14%)	18 (12.24%)	**.021**
**Outcome**	Discharged	140 (96.55%)	127 (86.39%)	**.004**
	Exitus	5 (3.45%)	20 (13.61%)	
**Length of stay**	Length of stay in ICU	11 (8-15)	8.5 (3-14)	.126
	Length of stay in hospital	11 (7-16)	11 (6-17)	.744
**Laboratory finding**	WBC (×1000)	5.58 (4.12-6.90)	6.24 (5.03-8.15)	**.001**
	Lymphocyte (×1000)	1.32 (0.96-1.83)	1.39 (0.95-1.84)	.871
	Hemoglobin	13.22 ± 1.74	13.55 ± 1.92	.124
	AST	23.5 (17.7-31)	23 (18-37)	.446
	ALT	20 (13-31)	19 (13-29)	.875
	CRP	9.6 (4-27.4)	19 (5.9-57.7)	**.001**

COPD, chronic obstructive pulmonary disease; CT, computed tomography; ICU, intensive care unit; WBC, white blood cell; AST, aspartate transaminase; ALT, alanine transaminase; *CRP, C-rea*ctive protein.

*P* < 0.05 values are statistically significant and also highlighted in bold.

**Table 3.** Gastrointestinal Symptoms

	**Diarrhea**			**Abdominal Pain**			**Nausea**			**Vomiting**		
	**Absent (n = 193)**	**Present (n = 99)**	***P***	**Absent (n = 241)**	**Present (n = 51)**	***P***	**Absent (n = 207)**	**Present (n = 85)**	***P***	**Absent (n = 238)**	**Present (n = 54)**	***P***
**Age**	54.35 ± 17.53	45.30 ± 13.49	**<.001**	52.27 ± 16.94	46.65 ± 15.46	**.030**	52.99 ± 16.68	47.14 ± 16.49	**.007**	51.27 ± 16.45	51.35 ± 18.45	.974
**Male**	99 (51.30%)	45 (45.45%)	.345	126 (52.28%)	18 (35.29%)	**.040**	112 (54.11%)	32 (37.65%)	**.011**	122 (51.26%)	22 (40.74%)	.213
**Female**	94 (48.70%)	54 (54.55%)		115 (47.72%)	33 (64.71%)		95 (45.89%)	53 (62.35%)		116 (48.74%)	32 (59.26%)	
**CT findings**	157 (81.35%)	75 (75.76%)	.334	195 (80.91%)	37 (72.55%)	.249	169 (81.64%)	63 (74.12%)	.198	187 (78.57%)	45 (83.33%)	.552
**Stay in ICU**	42 (21.76%)	17 (17.17%)	.441	50 (20.75%)	9 (17.65%)	.757	48 (23.19%)	11 (12.94%)	.069	48 (20.17%)	11 (20.37%)	1.000
**Intubated**	18 (9.33%)	6 (6.06%)	.461	20 (8.30%)	4 (7.84%)	1.000	21 (10.14%)	3 (3.53%)	.102	21 (8.82%)	3 (5.56%)	.587
**Discharged**	173 (89.64%)	94 (94.95%)	.189	219 (90.87%)	48 (94.12%)	.588	185 (89.37%)	82 (96.47%)	.082	216 (90.76%)	51 (94.44%)	.589
**Exitus**	20 (10.36%)	5 (5.05%)		.189	22 (9.13%)		3 (5.88%)	.588		22 (10.63%)	3 (3.53%)		.082	22 (9.24%)	3 (5.56%)	.589
**Length of stay in ICU (days)**	9.5 (3-15)	11 (8-14)	.261	9.5 (3-15)	13 (11-14)	.123	8.5 (3.5-14.5)	13 (11-15)	.215	9.5 (3.5-15)	11 (10-14)	.424
**Length of stay in hospital (days)**	11 (6-17)	11 (7-14)	.426	11 (6-17)	11 (6-15)	.685	11 (6-17)	11 (7-16)	.666	11 (6-16)	12 (9-17)	.059

CT, computed tomography; ICU, intensive care unit.

*P* < 0.05 values are statistically significant and also highlighted in bold.

## References

[1] Huang C Wang Y Li X et al. Clinical features of patients infected with 2019 novel coronavirus in Wuhan, China. The Lancet. 2020;395(10223):497 506. (10.1016/S0140-6736(20)30183-5) PMC7159299 31986264

[2] Klopfenstein T Kadiane-Oussou NJ Royer PY Toko L Gendrin V Zayet S . Diarrhea: an underestimated symptom in coronavirus disease 2019. Clin Res Hepatol Gastroenterol. 2020;44(3):282 283. (10.1016/j.clinre.2020.04.002) 32371006 PMC7183939

[3] Chen N Zhou M Dong X et al. Epidemiological and clinical characteristics of 99 cases of 2019 novel coronavirus pneumonia in Wuhan, China: a descriptive study. Lancet. 2020;395(10223):507 513. (10.1016/S0140-6736(20)30211-7) 32007143 PMC7135076

[4] Zhou F Yu T Du R et al. Clinical course and risk factors for mortality of adult inpatients with COVID-19 in Wuhan, China: a retrospective cohort study. Lanset. (10.1016/s0140-6736(20)30566-3) PMC7270627 32171076

[5] Pan L Mu M Yang P et al. Clinical characteristics of COVID-19 patients with digestive symptoms in Hubei, China: a descriptive, cross-sectional, multicenter study. Am J Gastroenterol. 2020;115(5):766 773. (10.14309/ajg.0000000000000620) 32287140 PMC7172492

[6] Ramachandran P Onukogu I Ghanta S et al. Gastrointestinal symptoms and outcomes in hospitalized coronavirus disease 2019 patients. Dig Dis. 2020;38(5):373 379. (10.1159/000509774) 32599601 PMC7445385

[7] Galanopoulos M Gkeros F Doukatas A et al. COVID-19 pandemic: pathophysiology and manifestations from the gastrointestinal tract. World J Gastroenterol. 2020;26(31):4579 4588. (10.3748/wjg.v26.i31.4579) 32884218 PMC7445869

[8] Ding S Liang TJ . Is SARS-CoV-2 also an enteric pathogen with potential fecal–oral transmission? A COVID-19 virological and clinical Review. Gastroenterology. 2020;159(1):53 61. (10.1053/j.gastro.2020.04.052) 32353371 PMC7184994

[9] Vaduganathan M Vardeny O Michel T McMurray JJV Pfeffer MA Solomon SD . Renin-angiotensin-aldosterone system inhibitors in patients with Covid-19. N Engl J Med. 2020;382(17):1653 1659. (10.1056/NEJMsr2005760) 32227760 PMC7121452

[10] Gui M Song W Zhou H et al. Cryo-electron microscopy structures of the SARS-CoV spike glycoprotein reveal a prerequisite conformational state for receptor binding. Cell Res. 2017;27(1):119 129. (10.1038/cr.2016.152) 28008928 PMC5223232

[11] Zhang H Kang Z Gong H et al. Digestive system is a potential route of COVID-19: an analysis of single-cell coexpression pattern of key proteins in viral entry process. Gut. 2020;69(6):1010 1018. (10.1136/gutjnl-2020-320953)

[12] Sungnak W Huang N Bécavin C et al. SARS-CoV-2 entry factors are highly expressed in nasal epithelial cells together with innate immune genes. Nat Med. 2020;26(5):681 687. (10.1038/s41591-020-0868-6) 32327758 PMC8637938

[13] Effenberger M Grabherr F Mayr L et al. Faecal calprotectin indicates intestinal inflammation in COVID-19. Gut. 2020;69(8):1543 1544. (10.1136/gutjnl-2020-321388) 32312790 PMC7211078

[14] Darnell ME Subbarao K Feinstone SM Taylor DR . Inactivation of the coronavirus that induces severe acute respiratory syndrome, SARS-CoV. J Virol Methods. 2004;121(1):85 91. (10.1016/j.jviromet.2004.06.006) 15350737 PMC7112912

[15] Xiao F Sun J Xu Y et al. Infectious SARS-CoV-2 in feces of patient with severe COVID-19. Emerg Infect Dis. 2020;26(8):1920 1922. (10.3201/eid2608.200681) 32421494 PMC7392466

[16] Wang W Xu Y Gao R et al. Detection of SARS-CoV-2 in different types of clinical specimens. JAMA. 2020;323(18):1843 1844. (10.1001/jama.2020.3786) 32159775 PMC7066521

[17] Wu Y Guo C Tang L et al. Prolonged presence of SARS-CoV-2 viral RNA in faecal samples. Lancet Gastroenterol Hepatol. 2020;5(5):434 435. (10.1016/S2468-1253(20)30083-2) 32199469 PMC7158584

[18] Wei XS Wang X Niu YR et al. Diarrhea is associated With prolonged symptoms and viral carriage in corona virus disease 2019. Clin Gastroenterol Hepatol. 2020;18(8):1753-1759.e2. (10.1016%2Fj.cgh.2020.04.030) 10.1016/j.cgh.2020.04.030 PMC7165091 32311512

[19] Cheung KS Hung IFN Chan PPY et al. Gastrointestinal manifestations of SARS-CoV-2 infection and virus load in fecal samples from a Hong Kong cohort: systematic review and meta-analysis. Gastroenterology. 2020;159(1):81 95. (10.1053/j.gastro.2020.03.065) 32251668 PMC7194936

[20] Brogna B Brogna C Petrillo M et al. SARS-CoV-2 detection in fecal sample from a patient with typical findings of COVID-19 pneumonia on CT but negative to multiple SARS-CoV-2 RT-PCR tests on oropharyngeal and nasopharyngeal swab samples. Medicina (Kaunas). 2021;57(3). (10.3390/medicina57030290) PMC8003654 33804646

[21] Bwire GM Majigo MV Njiro BJ Mawazo A . Detection profile of SARS-CoV-2 using RT-PCR in different types of clinical specimens: a systematic review and meta-analysis. J Med Virol. 2021;93(2):719 725. (10.1002/jmv.26349) 32706393 PMC7404904

[22] Gu J Han B Wang J . COVID-19: gastrointestinal manifestations and potential fecal-oral transmission. Gastroenterology. 2020;158(6):1518 1519. (10.1053/j.gastro.2020.02.054) 32142785 PMC7130192

[23] Chan JF Lau SK To KK Cheng VC Woo PC Yuen KY . Middle East respiratory syndrome coronavirus: another zoonotic Betacoronavirus causing SARS-like disease. Clin Microbiol Rev. 2015;28(2):465 522. (10.1128/CMR.00102-14) 25810418 PMC4402954

[24] Guan WJ Ni ZY Hu Y et al. Clinical characteristics of coronavirus disease 2019 in China. N Engl J Med. 2020;382(18):1708 1720. (10.1056/NEJMoa2002032) 32109013 PMC7092819

[25] Tariq R Saha S Furqan F Hassett L Pardi D Khanna S . Prevalence and mortality of COVID-19 patients with gastrointestinal symptoms: a systematic review and meta-analysis. Mayo Clin Proc. 2020;95(8):1632 1648. (10.1016/j.mayocp.2020.06.003) 32753138 PMC7284248

[26] Mao R Qiu Y He JS et al. Manifestations and prognosis of gastrointestinal and liver involvement in patients with COVID-19: a systematic review and meta-analysis. Lancet Gastroenterol Hepatol. 2020;5(7):667 678. (10.1016/S2468-1253(20)30126-6) 32405603 PMC7217643

[27] Shehab M Alrashed F Shuaibi S Alajmi D Barkun A . Gastroenterological and hepatic manifestations of patients with COVID-19, prevalence, mortality by country, and intensive care admission rate: systematic review and meta-analysis. BMJ Open Gastroenterol. 2021;8(1):e000571. (10.1136/bmjgast-2020-000571) PMC7934201 33664052

[28] Avcı E Ardahanlı İ Öztaş E Dişibeyaz S . Is there a relationship between gastrointestinal symptoms and disease course and prognosis in COVID-19? A single-center pilot study. The Turk J Acad Gastroenterol. 2020;19(3):103 108. (10.17941/agd.847338)

[29] Weibiao Z Kai Q MiaoZeng Y et al. Gastrointestinal symptoms are associated with severity of coronavirus disease 2019. Eur J Gastroenterol Hepatol. 2021. (10.1097/meg.0000000000002072) 33470700

